# Deep learning-based growth prediction for sub-solid pulmonary nodules on CT images

**DOI:** 10.3389/fonc.2022.1002953

**Published:** 2022-10-12

**Authors:** Ri-qiang Liao, An-wei Li, Hong-hong Yan, Jun-tao Lin, Si-yang Liu, Jing-wen Wang, Jian-sheng Fang, Hong-bo Liu, Yong-he Hou, Chao Song, Hui-fang Yang, Bin Li, Ben-yuan Jiang, Song Dong, Qiang Nie, Wen-zhao Zhong, Yi-long Wu, Xue-ning Yang

**Affiliations:** ^1^ Guangdong Lung Cancer Institute, Guangdong Provincial People’s Hospital, Guangdong Academy of Medical Sciences, Guangzhou, China; ^2^ Guangzhou Shiyuan Electronics Co., Ltd, Guangzhou, China; ^3^ Yibicom Health Management Center, CVTE, Guangzhou, China; ^4^ Automation Science and Engineering, South China University of Technology, Guangzhou, China

**Keywords:** sub solid pulmonary nodules, growth, mass, deep learning, radiomics

## Abstract

**Background:**

Estimating the growth of pulmonary sub-solid nodules (SSNs) is crucial to the successful management of them during follow-up periods. The purpose of this study is to (1) investigate the measurement sensitivity of diameter, volume, and mass of SSNs for identifying growth and (2) seek to establish a deep learning-based model to predict the growth of SSNs.

**Methods:**

A total of 2,523 patients underwent at least 2-year examination records retrospectively collected with sub-solid nodules. A total of 2,358 patients with 3,120 SSNs from the NLST dataset were randomly divided into training and validation sets. Patients from the Yibicom Health Management Center and Guangdong Provincial People’s Hospital were collected as an external test set (165 patients with 213 SSN). Trained models based on LUNA16 and Lndb19 datasets were employed to automatically obtain the diameter, volume, and mass of SSNs. Then, the increase rate in measurements between cancer and non-cancer groups was studied to evaluate the most appropriate way to identify growth-associated lung cancer. Further, according to the selected measurement, all SSNs were classified into two groups: growth and non-growth. Based on the data, the deep learning-based model (SiamModel) and radiomics model were developed and verified.

**Results:**

The double time of diameter, volume, and mass were 711 vs. 963 days (P = 0.20), 552 vs. 621 days (P = 0.04) and 488 vs. 623 days (P< 0.001) in the cancer and non-cancer groups, respectively. Our proposed SiamModel performed better than the radiomics model in both the NLST validation set and external test set, with an AUC of 0.858 (95% CI 0.786–0.921) and 0.760 (95% CI 0.646–0.857) in the validation set and 0.862 (95% CI 0.789–0.927) and 0.681 (95% CI 0.506–0.841) in the external test set, respectively. Furthermore, our SiamModel could use the data from first-time CT to predict the growth of SSNs, with an AUC of 0.855 (95% CI 0.793–0.908) in the NLST validation set and 0.821 (95% CI 0.725–0.904) in the external test set.

**Conclusion:**

Mass increase rate can reflect more sensitively the growth of SSNs associated with lung cancer than diameter and volume increase rates. A deep learning-based model has a great potential to predict the growth of SSNs.

## Introduction

Lung cancer is the leading cause of cancer death, with an estimated 1.8 million deaths (18.0%) worldwide in 2020 (1). However, low-dose computed tomography (LDCT) is an effective screening tool for reducing lung cancer mortality in high-risk individuals (2). With the popularization of the LDCT for lung cancer screening, the detection rate for pulmonary nodules, particularly sub-solid nodules (SSNs), has been significantly improved (3).

SSNs, which include both ground-glass (GGNs) and part-solid (PSNs) nodules, have a higher likelihood of malignancy than solid nodules regardless of size (4). Although SSNs have a good prognosis when treated early, they are at serious risk of overdiagnosis and overtreatment (5). Further, predicting the growth of SSNs is crucial to the successful management of SSNs during follow-up periods. Compared with diameter and volume, an increase in mass is an early indicator of growth. However, manual measurement of tumor quality requires many human resources and is difficult to carry out in routine clinical practice (6). Artificial intelligence (AI) has provided great improvements in cancer imaging (7). For example, many studies have used radiomics and deep learning to progress their fields (8–12). However, in the field of SSNs, little progress has been made with the use of AI or other automatic methods.

Therefore, the aim of this study is to investigate the most sensitive measurement of diameter, volume, and mass, using automatic methods, for identifying the growth of SSNs, and further, to establish a deep learning-based model to predict the growth of SSNs based on consecutive computed tomography (CT) scans to provide evidence for follow-up and treatment plans.

## Materials and methods

### Study protocol

We retrospectively analyzed sub-solid nodule cases from the National Lunch Screening Trial (NLST) (2) from August 2002 to December 2009, Yibicom Health Management Center from August 2017 to January 2022, and Guangdong Provincial People’s Hospital from July 2011 to September 2021. The inclusion criteria for Yibicom Health Management Center and Guangdong Provincial People’s Hospital were as follows: (a) 30 ≤ aged ≤80 years old; (b) underwent at least 2-year examination records with thin-section (2.5 mm) CT images; (c) at least one sub-solid nodule; and (d) the diameter 5 and 30 mm of the sub-solid nodule on initial CT images. The exclusion criteria were as follows: (a) only received one CT examination; (b) follow-up time was less than 2 years from the first CT examination; and (c) combined with other malignant tumors with history of less than 5 years, except for lung cancer. If the patient had multiple sub-solid nodules, the largest of the two nodules meeting the above conditions was selected for the study.

**Figure 1:**
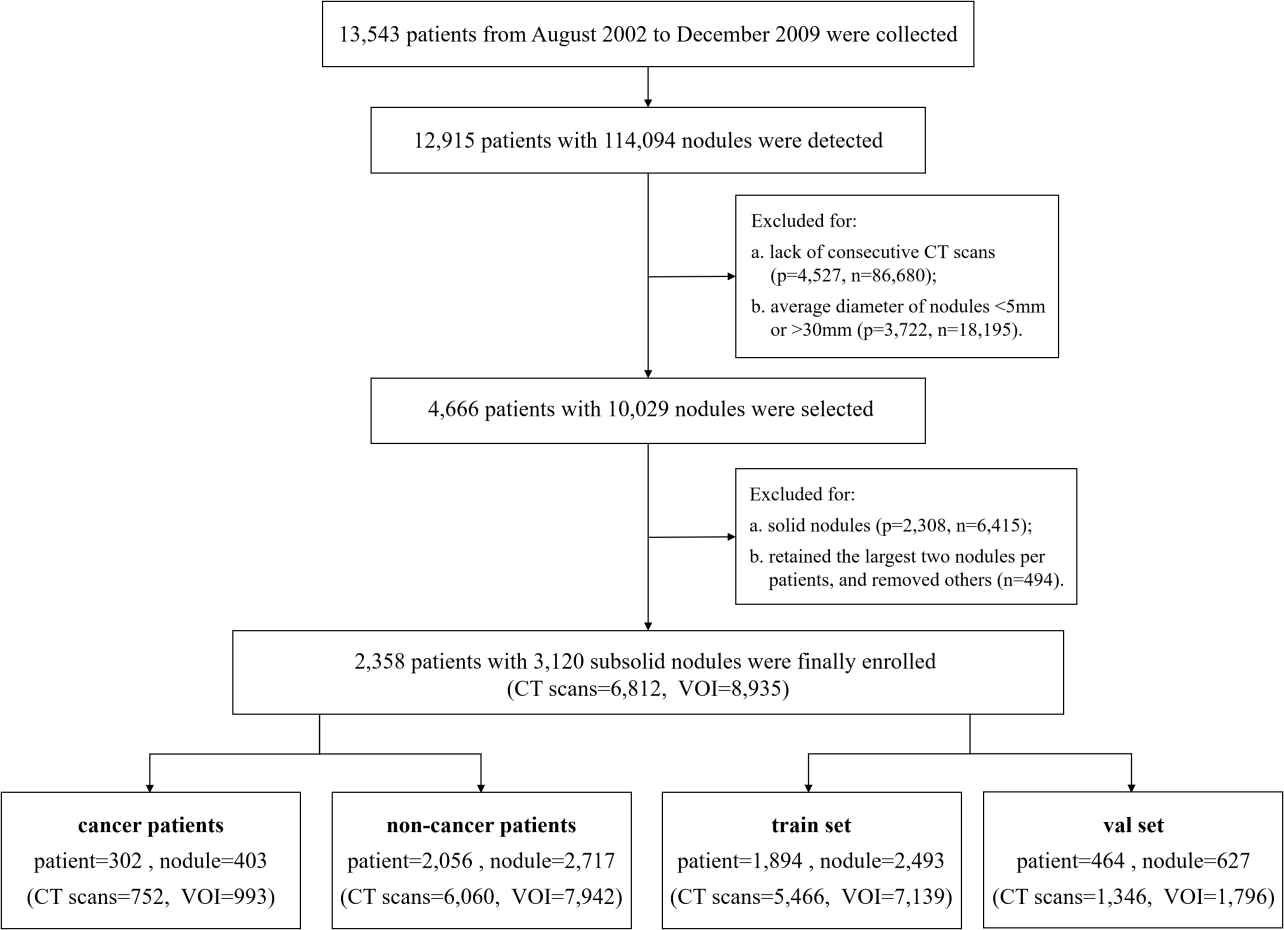
Flowchart of case selection on the NLST data set.

**Figure 2:**
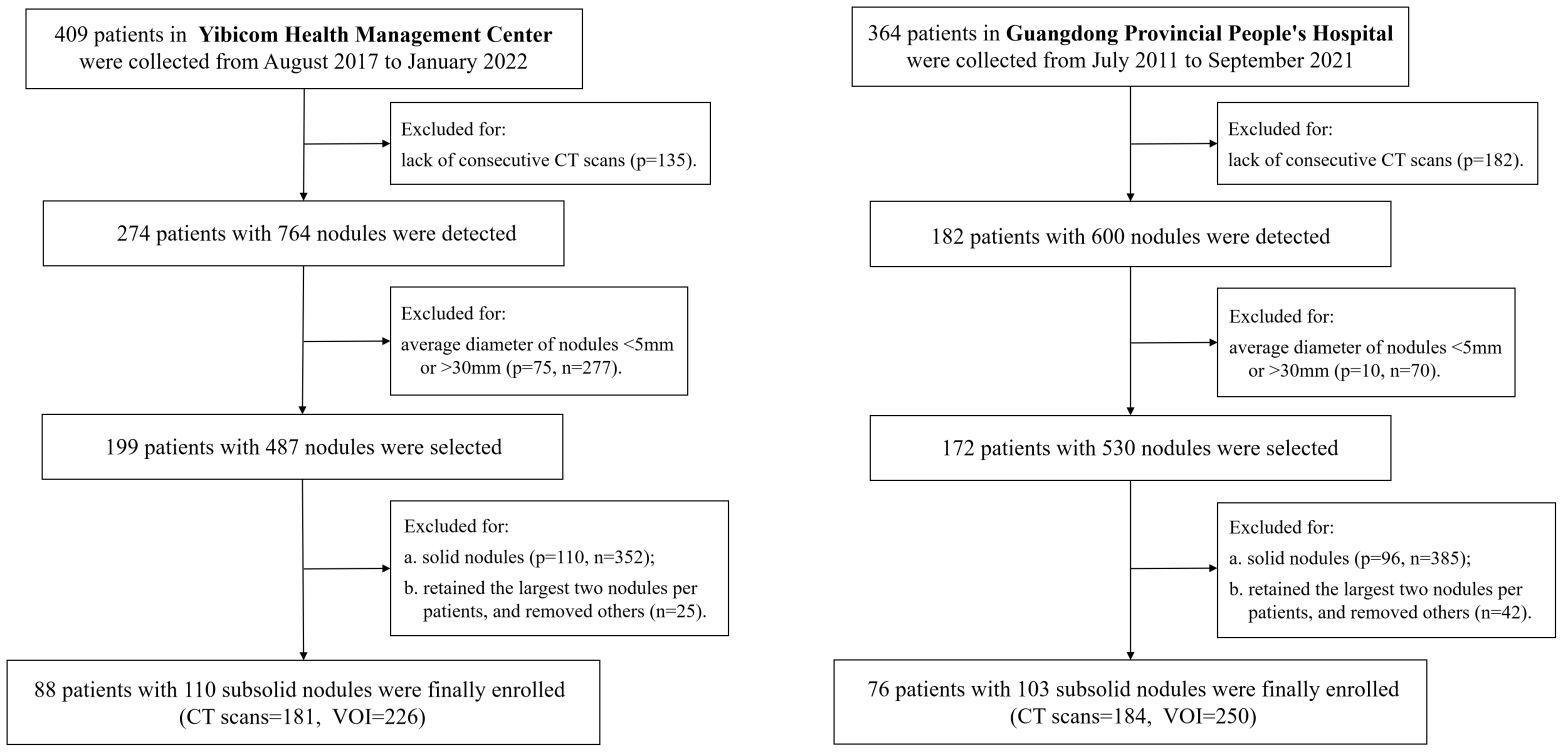
Flowchart of case selection on the external data set.

In total, 2,358 patients with 3,120 SSNs from the NLST dataset were enrolled and were randomly divided into the training set (1,894 patients with 2,493 SSNs) and validation set (464 patients with 627 SSNs), according to the ratio of 8:2 (**Figure 1**). In addition, 165 patients with 213 SSNs from Yibicom Health Management Center and Guangdong Provincial People’s Hospital were collected as an external test set (**Figure 2**).

### CT examinations

Axial images from 7,177 LDCT/CT examinations (6,812 CT examinations of 2,358 patients from NLST, 365 CT examinations of 165 patients from our two hospitals) were included in this study and were reconstructed by standard or lung kernel. If there were more than three available CT examinations, the latest three exams were enrolled. In total, 9,411 sub-solid nodule volumes from the axial images were extracted to assess the growth (**Figures 1**, **2**).

### Image analysis

**Figure 3:**
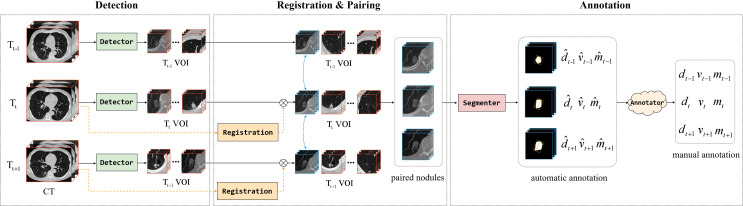
The pipeline of organizing the dataset, including CT scan registration, ROI pairing, and class annotation. The letters d, v, and m denote the diameter, volume, and mass of lung nodules, respectively.

In order to analyze the changes in diameter, volume, and mass of SSNs over consecutive years, we followed the method in Fang et al., to pair the same nodules between different CT scans (13). Our data organization approach aimed to ascertain the diameter, volume, and mass change of SSNs in consecutive CT scans (**Figure 3**). A semiautomatic pipeline was developed to process the consecutive CT scans. First, we detected and identified the SSNs on original CT scans. We then performed 3D image registration for the second (T_t_) and third (T_t+1_) CT scans in terms of the first scan (T_t−1_) and paired 3D volumes of interest (VOIs) containing SSNs to match the same sub-solid nodule at different time points. Next, we employed a segmenter to automatically crop out the lesion of nodules in VOIs to calculate their diameter, volume, and mass. After automatically annotating, we performed a manual review to acquire reliable labels.

Specifically, two popular CT datasets, Lung Nodule Analysis (LUNA)16 (14) and Lung Nodule Database (LNDb) (15), were used to train our models (detector and segmenter), in which the detector for VOI identification was a 3D variant of CenterNet (16) and the segmenter for lesion segmentation was a multi-scale 3D UNet (17). The results of the detector on LUNA16 were FROC = 0.966, recall = 0.978, and precision = 0.654. The segmenter has a dice of 0.838 on LUNA16.

### Growth measurement

There were three measurements for evaluating the growth of nodule, after semiautomatic acquisition of the nodule mask (1): the diameter, which was the longest side of the smallest circumscribed rectangle on the maximal surface of the nodule mask; (2) the volume (V in mm^3^), which was computed by multiplying the voxel number and the volume of a single voxel; and (3) the mass, which was computed as follows: M = V × (A + 1,000)/1,000 (18), where A is the average CT attenuation value (HU) and V is the volume of the nodule.

The SSNs from NLST were divided into cancer group and non-cancer group according to follow-up confirmation results. For each SSN, we calculated the increase rates of the diameter, volume, and mass during follow-up, respectively, then compared the three relative rates between the cancer group and non-cancer group to evaluate the most appropriate way for identifying the growth.

### Growth prediction model

According to the selected measurement, all SSNs were classified into two groups: growth group and non-growth group. The deep learning-based model and radiomics model were developed using the training set and were verified in the validation and test sets, respectively.

The radiomics model was the logistic regression model based on radiomics features, which was extracted from the shape and appearance of SSN in 3D VOIs and selected by the Least Absolute Shrinkage and Selection Operator (LASSO) (19). In total, 1,218 features were extracted and 60 features were selected for modeling the logistic regression model.

**Figure 4:**
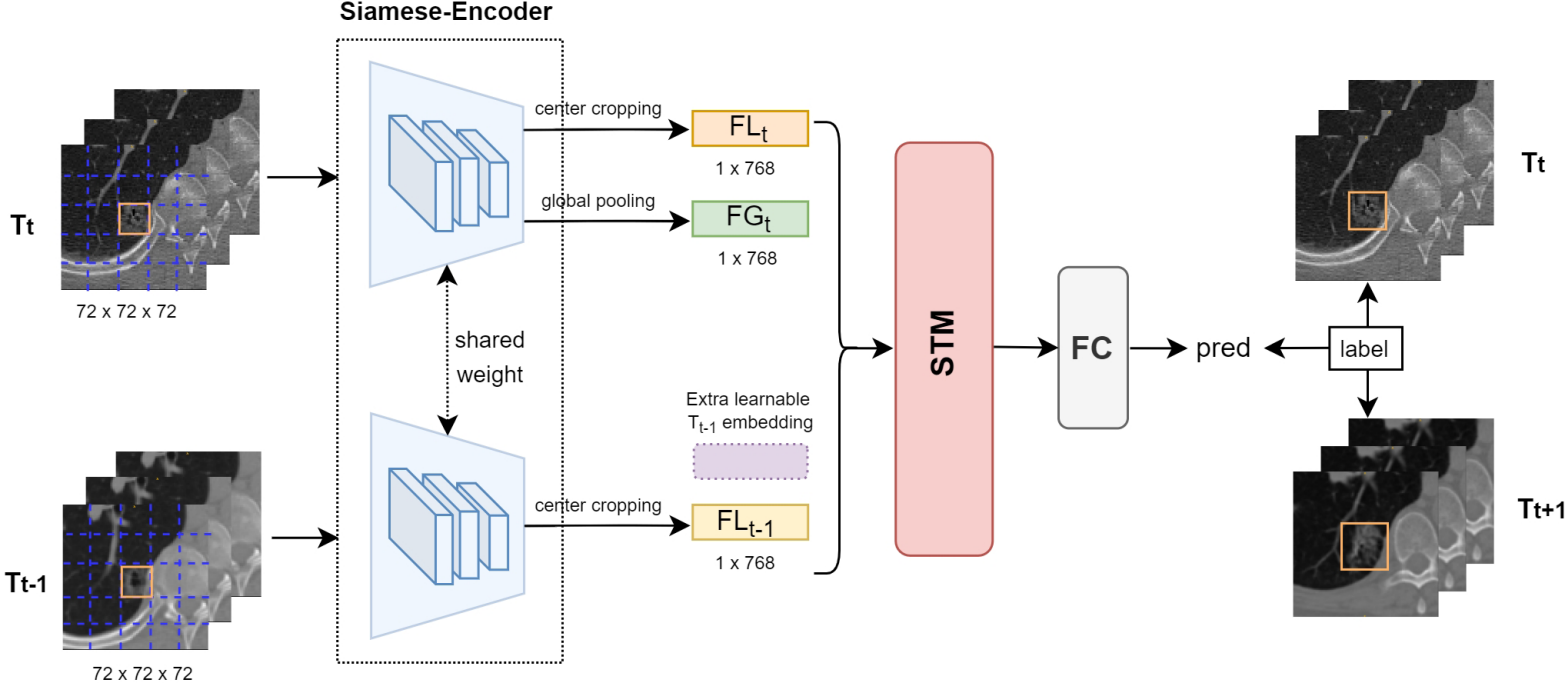
Overview of our proposed deep learning-based growth prediction model (called SiamModel), where FG_t_, FL_t_, and FL_t−1_ represent global feature embedding of T_t_ VOI, local feature embedding of T_t_ VOI, and local feature embedding of T_t−1_ VOI, respectively. The two encoders, whose backbone was ViT-B, in the Siamese-encoder shared weights.

Following selection (13), the deep learning-based model was identified (called SiamModel, **Figure 4**), where FG_t_, FL_t_, and FL_t−1_ represent global feature embedding of T_t_ VOI, local feature embedding of T_t_ VOI, and local feature embedding of T_t−1_ VOI, respectively. A learnable embedding FL_t−1_ was provided if T_t−1_ VOI was unavailable, which occurred when there were only two CT scans. For a given subject, the 3D VOI pairs (T_t−1_ and T_t_) taken from CT scans at sequential time points were fed into the Siamese encoder for extracting feature embedding. After fusing the features using the spatial-temporal mixer (STM) module (13), the fully connected layer was used to predict the growth probability. It was worth mentioning that the global information of VOIs was changeless on T_t−1_ and T_t_. Hence, we only learned global feature embedding from T_t_ without T_t−1_. However, the local information of the same nodule in T_t−1_ and T_t_ was different and highly discriminative for growth prediction. Therefore, we learned local feature embeddings from both T_t−1_ and T_t_ to capture the evolving local information.

To efficiently leverage changing information of SSNs in both non-growth and growth groups, we took the weighted smooth-L1 loss instead of cross-entropy loss to train our model, as follows:


(1) $$L=\alpha \times SmoothL_{1}(p,\;y)\times I_{\geq }+SmoothL_{1}(p,\;y)\times (1-I_{\geq })$$

In this model, p and y are the model output and ground truth of the relative growth rate, respectively. The indicator function Iy ≥ r = 1 if y ≥r, and 0 otherwise, and r was set to 0.1. α is the imbalance coefficient and was set to 1.0, 2.0, 3.0, 4.0, and 5.0 for our experiments, where we found that 3.0 was the best value.

The model was trained from scratch for 100 epochs with the AdamW optimizer (20), with a weight decay of 0.05 and a momentum of 0.9. The batch size was set as 16, and learning rate from 10e-6 to lr×Batchsize/64 in the first five epochs, where lr = 5e-4, and then scheduled by the cosine annealing strategy (20).

### Statistical analysis

Python 3.6.8 software with scipy.stats (1.8) and sklearn.metrics (1.0) packages was employed for data processing and statistical analysis. The reported statistical significance levels were all two-sided, and P< 0.05 was considered statistically significant.

Continuous variables were expressed as means ± standard deviations and compared with t-tests. Categorical variables were expressed by frequency and compared using the χ^2^ test. The discriminatory ability of these growth prediction models was evaluated with receiver operating characteristic (ROC) curves. Then, the non-parametric bootstrap was used to estimate the variability around each of the performance measures.

## Results

### Growth-to-variability ratio

In total, 3,120 SSNs from 2,358 patients in the NLST dataset, including 2,983 (96%) GGNs and 137 (4%) part-solid nodules (PSNs), were selected for the study. Most patients with a total of 2,695 SSNs received at least three CT scans. To evaluate the best way for identifying the sub-solid nodule growth, we divided those SSNs from NLST into the cancer group (403 SSNs) and non-cancer group (2,717 SSNs) according to follow-up confirmation results. The increase rate and doubling time (21) of diameter, volume, and mass were calculated respectively (**Table 1**). The P-values of the measurements (without diameter double time) compared between cancer and non-cancer groups were less than 0.05. In addition, mass had the smallest P-value, indicating that the difference in mass was more pronounced for cancer and non-cancer groups. In addition, mass had the shortest double time in the cancer group, which means mass has a better sensitivity for growth.

**Table 1:** Different rates of change (between the latest two consecutive examine) in the NLST dataset.

Type		Cancer	Non-cancer	P-value
Number**		187	2508	–
Diameter	Rate	0.07 ± 0.23	0.01 ± 0.16	0.002
	Double time*	711 (638, 894)	793 (716, 899)	0.2
Volume	Rate	0.49 ± 2.05	0.06 ± 0.66	0.005
	Double time*	552 (344, 725)	621 (458, 801)	0.04
Mass	Rate	0.74 ± 3.39	0.08 ± 0.83	0.009
	Double time*	488 (321, 630)	623 (463, 799)	<0.001

Double time* (Q1, Q3): selected within [1, 1,000] for statistics. Number**: SSNs with consecutive Tt−1, Tt, and Tt+1.

**Figure 5:**
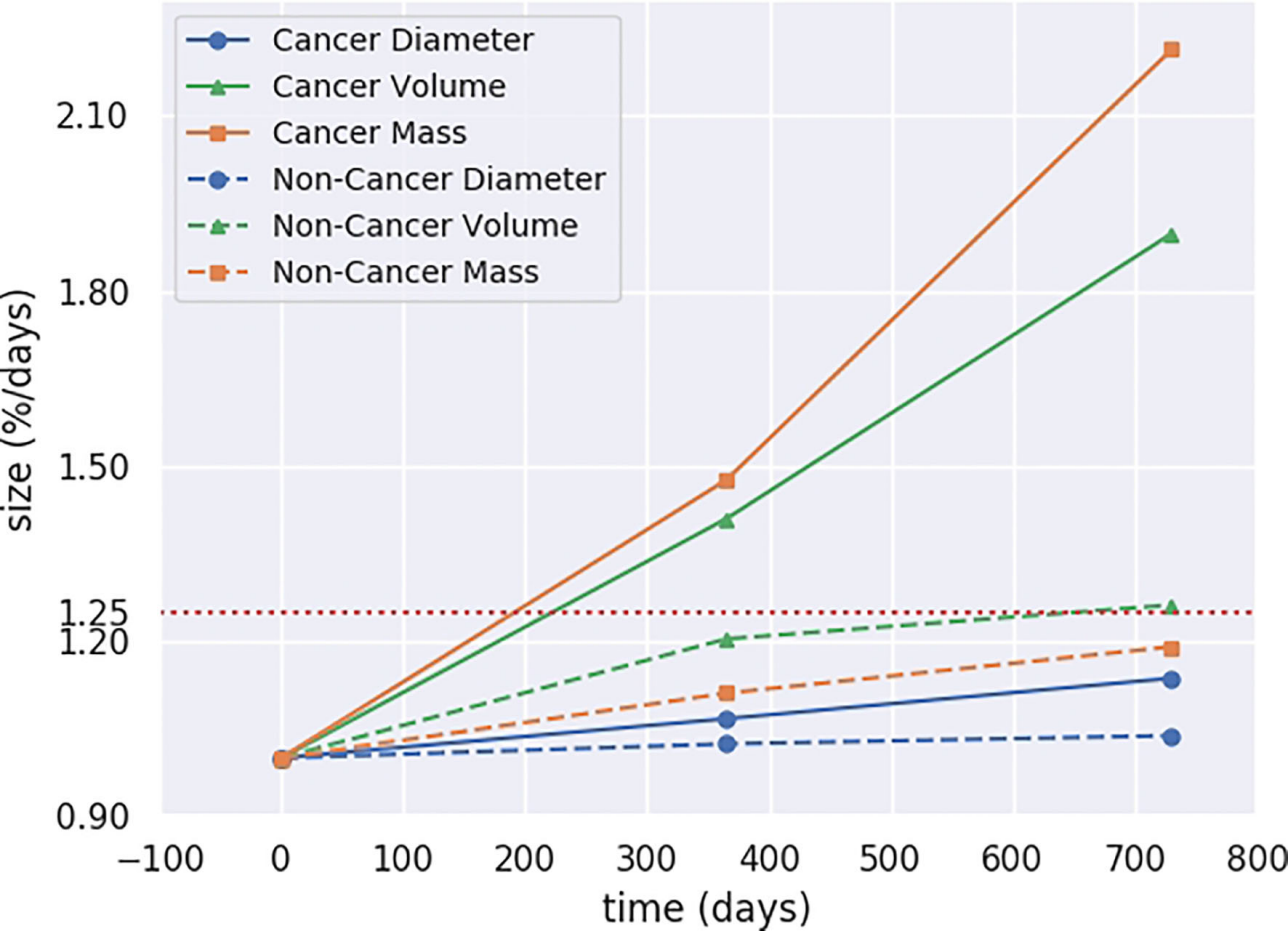
Progression in mass, volume, and diameter of SSNs.

The mean time between the first and last CT examinations of the selected SSNs was 739 days (range, 521–1,274 days). During this period, the diameter, volume, and mass of the SSNs in the cancer group increased with a mean of 14%, 90%, and 121%, respectively, while in the non-cancer group the mean increased at 4%, 26%, and 19%, respectively. For distinguishing growth in the cancer and non-cancer groups, the increase rate in mass was more significant than those in volume and diameter (**Figure 5**). When the mass increased at greater than 25%, the SSNs showed a significant growth trend and were more likely to deteriorate into lung cancer.

### Growth and non-growth subsolid nodule characteristics

According to the above analysis, the growth of SSNs was defined as an increase in mass at 25% within 1 year. There were 2,493 SSNs (174 growth, 2,319 non-growth), 627 SSNs (38 growth, 589 non-growth), and 213 SSNs (9 growth, 204 non-growth) in the training, validation, and external test sets, respectively (**Table 2**). In the training and validation sets from NLST, there were significant differences in average CT value (P< 0.01) and diameter (P< 0.001) between the growth and non-growth groups. However, in the external test set, only diameter (P = 0.04) was significantly different between the growth and non-growth groups.

**Table 2:** The characteristics of patients and sub-solid nodules and the results of univariate analysis on training, validation, and external test sets.

Dataset	Characteristics	Growth	Non-growth	*T/χ* ^2^ value	*P* value
		(*Q*_1_, *Q*_3_)	(*Q*_1_, *Q*_3_)		
	Number	174	2319	–	–
	Age (years)	63 (59, 67)	62 (58, 66)	1.48	0.14
	Gender (male/female)	102*/*72	1312*/*1007	0.2	0.66
NLST train set	Average CT value (HU)	-598.4 (-692.1, 538.7)	-640.6 (-703.3, -589.7)	4.1	*<*0.001
	Diameter (mm)	9.6 (5, 12.2)	5.6 (4, 6)	8.1	*<*0.001
	Texture (GGNs*/*PSNs)	142*/*32	2247*/*72	90.82	0
	Number	38	589	–	–
	Age (years)	65 (61, 69)	62 (58, 66)	3.6	*<*0.001
	Gender (male*/*female)	19/19	363/226	1.57	0.21
NLST Val Set	Average CT value (HU)	-564.2 (-679.8, -454.1)	-630.1 (-684.3, -581.1)	2.72	*<*0.01
	Diameter (mm)	12.9 (5.8, 20.5)	5.6 (4.0, 6.4)	6.03	*<*0.001
	Texture (GGNs*/*PSNs)	19/19	363/226	31.6	0
	Number	9	204	–	–
	Age (years)	58 (47, 67)	53 (46, 61)	0.83	0.43
	Gender (male*/*female)	4/5	84/120	0.02	0.88
External test set	Average CT value (HU)	-553.2 (-700.1, -460.9)	-657.5 (-723.2, 600.9)	1.51	0.17
	Diameter(mm)	9.6 (7.1, 12)	6.2 (4, 7.1)	2.47	0.04
	Texture (GGNs*/*PSNs)	8/1	202/2	1.16	0.28

### Model performance comparison

The deep learning-based and radiomics models were developed using the training set and were verified in the validation and test sets. The AUC of SiamModel was 0.858 (95% CI 0.786–0.921) in the validation set and 0.862 (95% CI 0.789–0.927) in the external test set (**Table 3**). The comparable results between the validation and external test sets showed that SiamModel had good generalization ability.

**Table 3:** Performance of different models using only one CT scan in the validation and external test sets.

Data set	Method	AUC	Sensitivity	Specificity	PPV	NPV
		(95% CI)	(95% CI)	(95% CI)	(95% CI)	(95% CI)
NLST val	SiamModel (our)	0.858	0.632	0.921	0.341	0.975
		(0.786-0.921)	(0.485-0.786)	(0.898-0.942)	(0.239-0.456)	(0.961-0.987)
	SiamModel (once, our)	0.855	0.843	0.651	0.136	0.985
		(0.793-0.908)	(0.724-0.952)	(0.613-0.692)	(0.094-0.184)	(0.973-0.995)
	STM[13]	0.823	0.764	0.738	0.157	0.98
		(0.731-0.898)	(0.622-0.897)	(0.702-0.774)	(0.109-0.217)	(0.965-0.991)
	Radiomics (once)	0.76	0.763	0.465	0.085	0.968
		(0.646-0.857)	(0.615-0.889)	(0.424-0.506)	(0.059-0.114)	(0.947-0.986)
External test	SiamModel (our)	0.862	0.893	0.749	0.134	0.994
		(0.789-0.927)	(0.625-1.000)	(0.685-0.807)	(0.056-0.230)	(0.980-1.000)
	SiamModel (once, our)	0.821	0.889	0.669	0.106	0.993
		(0.725-0.904)	(0.625-1.000)	(0.608-0.731)	(0.040-0.179)	(0.977-1.000)
	STM[13]	0.806	0.895	0.574	0.083	0.992
		(0.693-0.902)	(0.636-1.000)	(0.507-0.664)	(0.034-0.141)	(0.972-1.000)
	Radiomics (once)	0.681	0.663	0.528	0.059	0.972
		(0.506-0.841)	(0.333-1.000)	(0.461-0.599)	(0.019-0.107)	(0.939-1.000)

Compared with STM (13), our SiamModel obtained better performance with the AUC of 0.858 (95% CI 0.786–0.921) vs. 0.823 (95% CI 0.731–0.898) in the validation set and 0.862 (95% CI 0.789–0.927) vs. 0.806 (95% CI 0.693–0.902) in the external test set, which indicated the superiority of our proposed weighted smooth-l1 loss for SSN growth prediction.

**Figure 6:**
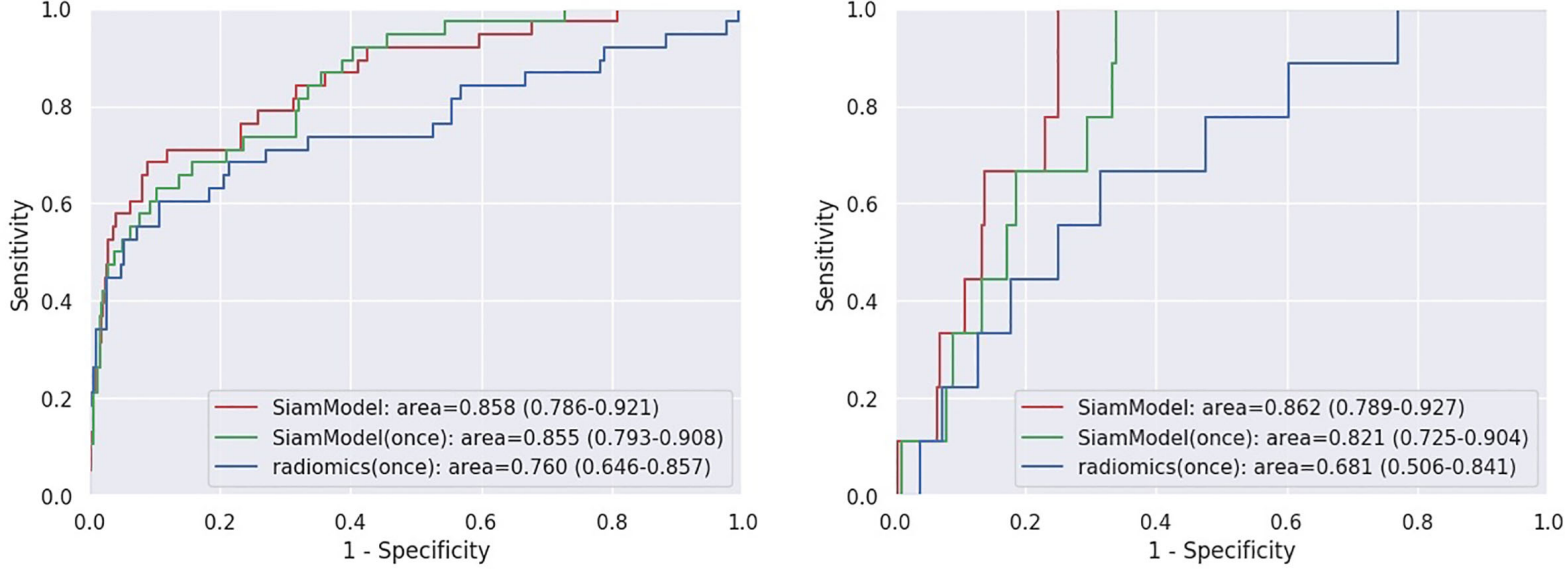
Receiver operating characteristic (ROC) curves on NLST validation set (left) and external test set (right). SiamModel (once) and radiomics (once) are only using Tt VOI as input.

Assuming all T_t−1_ scans were unavailable in datasets (using only T_t_ VOI as input), the performance of SiamModel and radiomics model was compared as shown in **Table 3** and **Figure 6**. In the NLST validation set, the AUC values of SiamModel and radiomics model were 0.855 (95% CI 0.793–0.908) and 0.760 (95% CI 0.646–0.857), respectively, and 0.821 (95% CI 0.725–0.904) and 0.681 (95% CI 0.506–0.841) in the external test set. Therefore, our SiamModel performed better than the radiomics model in both the NLST validation set and external test set (**Figure 6**).

Comparing one using only T_t_ VOI as input, we saw that our SiamModel with two VOIs (T_t−1_ and T_t_) as input could perform slightly better, with an AUC of 0.858 vs. 0.855 in the NLST validation set and 0.862 vs. 0.821 in the external test set.

### Examples of model prediction

**Figure 7:**
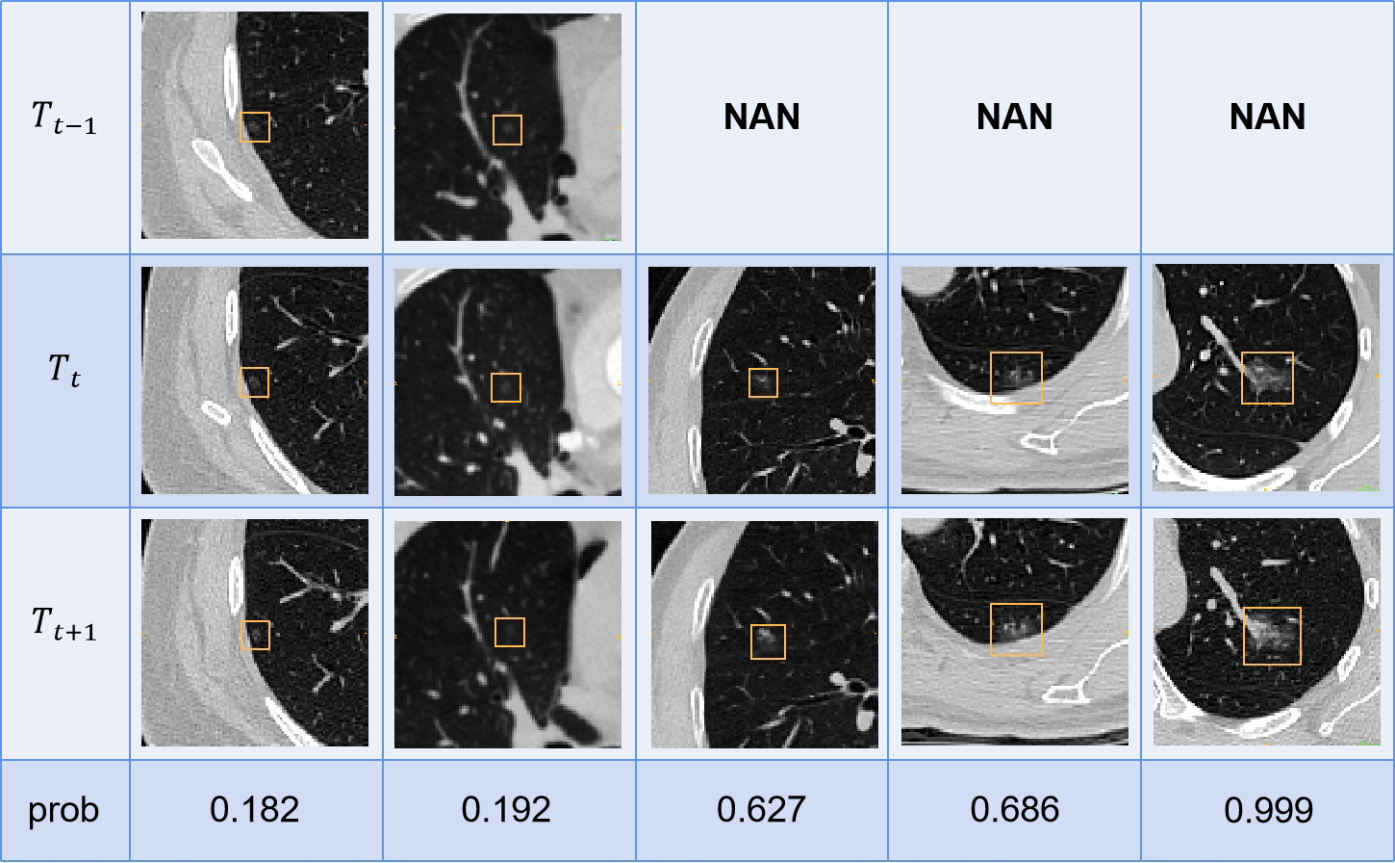
Examples of predicting the growth probability by our method in the external test set.

**Figure 7** provides examples predicted by our SiamModel in the external test set.

The probability of growth was calculated as shown in Eq. 2, where p and th are the model output and threshold selected for sensitivity and specificity as shown in **Table 3**, respectively, and τ is the sharpening coefficient, set as 0.1 to map p to 0-1:


(2) $$prob=\frac{e(p-th)/\tau }{1+e(p-th)/\tau }$$

The predicted result with prob ≥0.5 indicated the growth, which required more attention from doctors and relatively intensive follow-up.

## Discussion

Our study first compared the effectiveness of diameter, volume, and mass for assessing SSN growth based on the big data from NLST and found that mass had a better sensitivity to assess SSN growth. Then, we developed the deep learning-based model (SiamModel) to predict the mass growth of SSNs and achieved good performance in both the validation set (AUC = 0.858) and the external test set (AUC = 0.862).

For pulmonary nodules discovered in screening or incidentally, the first task was to assess risk of malignancy. There are several models (22–24) which combined clinical and radiographic factors to estimate the malignancy probability and achieve fair performance (25). For those indeterminate nodules, follow-up would be recommended (26). The growth pattern of lung nodules (18, 27) would increase the accuracy rate to diagnose malignant nodules and reduce the false positives, although some benign nodules would grow (28).

In addition, there are several methods to measure nodule growth, such as diameter, volume, and mass. Nodule growth or the solid component in part-solid nodule growth is defined as an increase in diameter of more than 1.5 mm in Lung-Reporting and Data Systems (RADS) (27) and in the National Comprehensive Cancer Network (NCCN) algorithm (NCCN 2022 screening). While 2 mm was chosen as the threshold for defining growth in both overall nodule size and the solid component of a part-solid nodule in the Fleischner Society (24), I-ELCAP requires different sizes of growth according to initial diameter of nodules (I-ELCAP protocol. Available at https://www.ielcap.org/protocols Accessed June 14,2022). Because the investigator of NELSON thought volume measurements are more accurate than size by means of semiautomatic software calculation, growth was defined as a change in volume of at least 25% growth of pulmonary nodules (29, 30) and volume measurement was also recommended in the British Thoracic Society pulmonary nodule management guidelines (22). Additionally, the volume doubling time (VDT) of SSN would be longer, and VDT may not be sensitive to different indolent lung cancers from benign nodules. Our study also showed a mean of VDT cancer growth and non-cancer growth (552 vs. 621 days, P = 0.04) and was close to the NLST databases.

Furthermore, Hoop et al. (6) compared measurements of diameter, volume, and mass in 52 pulmonary GGNs and found that mass is the best method for identifying malignant GGNs and detection of growth of GGNs. Our study also found that mass growth was the most sensitive method to identify the growth of SSNs, since the growth of mass might reflect the volume, density, or solid component growth. Compared with Hoop et al.’s study (6), we investigated growth rate in SSNs from NLST and validated the data in a larger number of patients. Moreover, we used an automatic machine learning method to measure volume and mass, which would reduce the consumption of time and labor.

Initial size (31), CT attenuation (3), and history of lung cancer (30) were associated with GGN growth according to the first-time CT results and clinical factors. For those pulmonary nodules which require follow-up, previous studies focus on classifying and few rely on prediction factors for the GGO growth. Radiomics and deep learning techniques have been investigated to detect, segment, and classify in the field of pulmonary nodule management (7, 32) in the past 10 years. Radiomics could extract the high quantitative image features from medical imaging (32, 33) and build high-performance models with limited datasets. Several studies (34) have demonstrated that radiomic signatures can differentiate malignant and benign nodules with a sensitivity ranging from 76.2 to 92.9% and a specificity ranging from 72.7 to 96.1%. The combined radiographic factor or supervised machine learning with the radiomics model could achieve better performance (34, 35). In the traditional radiomics methods to classify pulmonary nodules (11), large amounts of labor are required for manual tumor segmentation and feature extraction. Deep learning algorithms could detect and segment pulmonary nodules automatically and build predictive models. Ardila et al. (8) developed a predictive model of the risk of lung cancer by the 3D deep learning method and achieved algorithms that were comparable to, or could even outperform, radiologists with or without prior CT imaging. A Lung Cancer Prediction Convolutional Neural Network model (36) was also found to outperform the Brock model to predict risk of lung cancer. A deep machine learning algorithm developed by Huang et al. (21) was compared with Lung-RADS and volume doubling time to inform lung cancer incidence with 1, 2, and 3 years. As for growth of pulmonary nodules, Tao et al. (18) first manually segmented 313 lung nodules in 246 patients in their hospital then developed a convolutional neural network (CNN) to model the imagery change in the nodules from the baseline CT image to the follow-up CT image and achieved an AUC of 0.857 for solid nodules and 0.843 for GGNs in differentiating growth and non-growth nodules. Compared with Tao et al. (18), we first trained the detection and segmentation models on LUNA16 and LNDb datasets and then used them to identify and segment SSNs automatically which was easily reproducible. As for the growth of SSNs, we developed a deep learning-based model, called SiamModel. In the independent external test set, our SiamModel could predict the growth of SSNs with good performance (AUC = 0.862) and showed a significant improvement, compared with the radiomics model.

This study has the following limitations: (a) the growth and non-growth SSNs were extremely imbalanced in our external test set, so a validation bias might exist; and (b) the training set only contained low-dose CT scans, but the external set enrolled both normal CT and low-dose CT scans, therefore, it is still necessary to expand the training and test sets with more normal CT scans in further study; and (c) we defined an increase of at least 25% in mass as the growth of SSNs without an exact derivation which should be tested in further clinical practice.

Mass increase rate can reflect the growth of SSNs associated with lung cancer more sensitively than diameter and volume increase rates. Further, we established a deep learning-based model (called SiamModel) that could better predict the growth of SSNs on the base of mass, compared with the radiomics model.

## Data availability statement

The original contributions presented in the study are included in the article/supplementary material. Further inquiries can be directed to the corresponding authors.

## Ethics statement

This study was reviewed and approved by Guangdong Provincial People’s Hospital (KY-Z-2022-125-01). Written informed consent for participation was not required for this study in accordance with the national legislation and the institutional requirements.

## Author contributions

XY contributed to conception and design of the study. RL wrote the first draft of the manuscript and analyze data. AL organized the database. HHY performed the statistical analysis. JL, SL, BJ, SD, QN, WZ contributed to collect clinical data. BL, YW, JF, HL, YH, CS, HFY contributed to analyze data. YW supervised the study. All authors contributed to manuscript revision, read, and approved the submitted version.

## Funding

This work was supported by the research fund from Guangzhou Municipal Science and Technology Bureau (grant no. 202201011664 to XY).

## Conflict of interest

XY, YW, RL, HHY, JL, SL, BJ, SD, QN, WZ are employed by Guangdong Provincial People’s Hospital. BL is employed by South China University of Technology. Authors AL, JW, JF and HL are employed by Guangzhou Shiyuan Electronics Co., Ltd.

The remaining authors declare that the research was conducted in the absence of any commercial or financial relationships that could be construed as a potential conflict of interest.

## Publisher’s note

All claims expressed in this article are solely those of the authors and do not necessarily represent those of their affiliated organizations, or those of the publisher, the editors and the reviewers. Any product that may be evaluated in this article, or claim that may be made by its manufacturer, is not guaranteed or endorsed by the publisher.

## References

[1] Sung H Ferlay J Siegel RL Laversanne M Soerjomataram I Jemal A. Global cancer statistics 2020: Globocan estimates of incidence and mortality worldwide for 36 cancers in 185 countries. CA Cancer J Clin (2021) 71:209–49. doi: 10.3322/caac.21660 33538338

[2] Aberle DR Adams AM Berg CD Black WC Clapp JD Fagerstrom RM. Reduced lung-cancer mortality with low-dose computed tomographic screening. N Engl J Med (2011) 365:395–409. doi: 10.1056/NEJMoa1102873 21714641 PMC4356534

[3] Gao C Li J Wu L Kong D Xu M Zhou C. The natural growth of subsolid nodules predicted by quantitative initial ct features: A systematic review. Front Oncol (2020) 10:318. doi: 10.3389/fonc.2020.00318 32292716 PMC7119340

[4] Singh R Kalra MK Homayounieh F Nitiwarangkul C McDermott S Little BP. Artificial intelligence-based vessel suppression for detection of sub-solid nodules in lung cancer screening computed tomography. Quant Imaging Med Surg (2021) 11:1134–43. doi: 10.21037/qims-20-630 PMC7930659 33816155

[5] Sun Q Huang Y Wang J Zhao S Zhang L Tang W. Applying CT texture analysis to determine the prognostic value of subsolid nodules detected during low-dose CT screening. Clin Radiol (2019) 74:59–66. doi: 10.1016/j.crad.2018.07.103 30501892

[6] de Hoop B Gietema H van de Vorst S Murphy K van Klaveren RJ Prokop M. Pulmonary ground-glass nodules: increase in mass as an early indicator of growth. Radiology (2010) 255:199–206. doi: 10.1148/radiol.09090571 20123896

[7] Bi WL Hosny A Schabath MB Giger ML Birkbak NJ Mehrtash A. Artificial intelligence in cancer imaging: Clinical challenges and applications. CA Cancer J Clin (2019) 69:127–57. doi: 10.3322/caac.21552 PMC6403009 30720861

[8] Ardila D Kiraly AP Bharadwaj S Choi B Reicher JJ Peng L. End-to-end lung cancer screening with three-dimensional deep learning on low-dose chest computed tomography. Nat Med (2019) 25:954–61. doi: 10.1038/s41591-019-0447-x 31110349

[9] Ather S Kadir T Gleeson F. Artificial intelligence and radiomics in pulmonary nodule management: current status and future applications. Clin Radiol (2020) 75:13–9. doi: 10.1016/j.crad.2019.04.017 31202567

[10] Li R Xiao C Huang Y Hassan H Huang B. Deep learning applications in computed tomography images for pulmonary nodule detection and diagnosis: A review. Diagno (Basel) (2022) 12(2):298. doi: 10.3390/diagnostics12020298 PMC8871398 35204388

[11] Fahmy D Kandil H Khelifi A Yaghi M Ghazal M Sharafeldeen A. How ai can help in the diagnostic dilemma of pulmonary nodules. Cancers (Basel) (2022) 14(7):1840. doi: 10.3390/cancers14071840 35406614 PMC8997734

[12] Murchison JT Ritchie G Senyszak D Nijwening JH van Veenendaal G Wakkie J. Validation of a deep learning computer aided system for CT based lung nodule detection, classification, and growth rate estimation in a routine clinical population. PloS One (2022) 17:e0266799. doi: 10.1371/journal.pone.0266799 35511758 PMC9070877

[13] Fang J Wang J Li A Yan Y Hou Y Song C. Siamese Encoder- based spatial-temporal mixer for growth trend prediction of lung nodules on ct scans. arXiv (2022) 2206:03049. doi: 10.1007/978-3-031-16431-6_46

[14] Setio AAA Traverso A de Bel T Berens MSN Bogaard CVD Cerello P. Validation, comparison, and combination of algorithms for automatic detection of pulmonary nodules in computed tomography images: The LUNA16 challenge. Med Image Anal (2017) 42:1–13. doi: 10.1016/j.media.2017.06.015 28732268

[15] Pedrosa J Aresta G Ferreira C Rodrigues M Leit˜ao P Carvalho AS LNDb: A lung nodule database on computed tomography. arXiv (2019). doi: 10.48550/arXiv.1911.08434

[16] Zhou X Wang D Kr¨ahenbu¨hl P. Objects as points. arXiv (2019) 1904:07850. doi: 10.48550/arXiv.1904.07850

[17] Kushnure DT Talbar SN. MS-UNet: A multi-scale UNet with feature recalibration approach for automatic liver and tumor segmentation in CT images. Comput Med Imaging Graph (2021) 89:101885. doi: 10.1016/j.compmedimag.2021.101885 33684731

[18] Tao G Zhu L Chen Q Yin L Li Y Yang J. Prediction of future imagery of lung nodule as growth modeling with follow-up computed tomography scans using deep learning: a retrospective cohort study. Transl Lung Cancer Res (2022) 11:250–62. doi: 10.21037/tlcr-22-59 PMC8902095 35280310

[19] Tibshirani R. Regression shrinkage and selection *via* the lasso. J R Stat Soc: Ser B (Methodological) (1996) 58:267–88. doi: 10.1111/j.2517-6161.1996.tb02080.x

[20] Loshchilov I Hutter F. Stochastic gradient descent with warm restarts. Pro- ceedings 5th Int Conf Learn Represent (2016). 1–16. Available at: https://arxiv.org/abs/1608.03983v1

[21] Huang P Lin CT Li Y Tammemagi MC Brock MV Atkar-Khattra S. Prediction of lung cancer risk at follow-up screening with low-dose CT: a training and validation study of a deep learning method. Lancet Digit Health (2019) 1:e353–e62. doi: 10.1016/S2589-7500(19)30159-1 PMC7450858 32864596

[22] Baldwin DR Callister ME. The British thoracic society guidelines on the investigation and management of pulmonary nodules. Thorax (2015) 70:794–8. doi: 10.1136/thoraxjnl-2015-207221 26135833

[23] Gould MK Ananth L Barnett PG. A clinical model to estimate the pretest probability of lung cancer in patients with solitary pulmonary nodules. Chest (2007) 131:383–8. doi: 10.1378/chest.06-1261 PMC3008547 17296637

[24] Bankier AA MacMahon H Goo JM Rubin GD Schaefer-Prokop CM Naidich DP. Recommendations for measuring pulmonary nodules at ct: A statement from the fleischner society. Radiology (2017) 285:584–600. doi: 10.1148/radiol.2017162894 28650738

[25] Zhang K Wei Z Nie Y Shen H Wang X Wang J. Comprehensive analysis of clinical logistic and machine learning-based models for the evaluation of pulmonary nodules. JTO Clin Res Rep (2022) 3:100299. doi: 10.1016/j.jtocrr.2022.100299 35392654 PMC8980995

[26] Wood DE Kazerooni EA Aberle D Berman A Brown LM Eapen GA. NCCN guidelines® insights: Lung cancer screening, version 1.2022 (2022). Available at: https://jnccn.org/view/journals/jnccn/20/7/article-p754.xml. 10.6004/jnccn.2022.0036 35830884

[27] Pinsky PF Gierada DS Black W Munden R Nath H Aberle D. Performance of lung-rads in the national lung screening trial: A retrospective assessment. Ann Intern Med (2015) 162:485–91. doi: 10.7326/M14-2086 PMC4705835 25664444

[28] Zhang R Tian P Qiu Z Liang Y Li W. The growth feature and its diagnostic value for benign and malignant pulmonary nodules met in routine clinical practice. J Thorac Dis (2020) 12:2019–30. doi: 10.21037/jtd-19-3591 PMC7330364 32642104

[29] van Klaveren RJ Oudkerk M Prokop M Scholten ET Nackaerts K Vernhout R. Management of lung nodules detected by volume CT scanning. N Engl J Med (2009) 361:2221–9. doi: 10.1056/NEJMoa0906085 19955524

[30] de Koning HJ van der Aalst CM de Jong PA Scholten ET Nackaerts K Heuvelmans MA. Reduced lung-cancer mortality with volume ct screening in a randomized trial. N Engl J Med (2020) 382:503–13. doi: 10.1056/NEJMoa1911793 31995683

[31] Hiramatsu M Inagaki T Inagaki T Matsui Y Satoh Y Okumura S. Pulmonary ground-glass opacity (GGO) lesions-large size and a history of lung cancer are risk factors for growth. J Thorac Oncol (2008) 3:1245–50. doi: 10.1097/JTO.0b013e318189f526 18978558

[32] Lambin P Leijenaar RTH Deist TM Peerlings J de Jong EEC van Timmeren J. Radiomics: the bridge between medical imaging and personalized medicine. Nat Rev Clin Oncol (2017) 14:749–62. doi: 10.1038/nrclinonc.2017.141 28975929

[33] Lambin P Rios-Velazquez E Leijenaar R Carvalho S van Stiphout RG Granton P. Radiomics: extracting more information from medical images using advanced feature analysis. Eur J Cancer (2012) 48:441–6. doi: 10.1016/j.ejca.2011.11.036 PMC4533986 22257792

[34] Wu YJ Wu FZ Yang SC Tang EK Liang CH. Radiomics in early lung cancer diagnosis: From diagnosis to clinical decision support and education. Diagno (Basel) (2022) 12(5):1064. doi: 10.3390/diagnostics12051064 PMC9139351 35626220

[35] Tan M Ma W Sun Y Gao P Huang X Lu J. Prediction of the growth rate of early-stage lung adenocarcinoma by radiomics. Front Oncol (2021) 11:658138. doi: 10.3389/fonc.2021.658138 33937070 PMC8082461

[36] Massion PP Antic S Ather S Arteta C Brabec J Chen H. Assessing the accuracy of a deep learning method to risk stratify indeterminate pulmonary nodules. Am J Respir Crit Care Med (2020) 202:241–9. doi: 10.1164/rccm.201903-0505OC PMC7365375 32326730

